# Sleep quality after shoulder arthroplasty – A systematic review

**DOI:** 10.1177/17585732261450975

**Published:** 2026-05-23

**Authors:** Kalter Hali, Marc A Manzo, Sean Mckellar, Matthew J Raleigh, Ujash Sheth

**Affiliations:** 1 Division of Orthopaedic Surgery, 7938 University of Toronto, Toronto, Ontario, Canada; 2 Division of Orthopaedic Surgery, 71545 Sunnybrook Health Sciences Centre, Sunnybrook Orthopaedic Upper Limb, Toronto, Ontario, Canada

**Keywords:** Shoulder arthroplasty, sleep quality, actigraphy

## Abstract

**Background:**

Shoulder arthroplasty has been shown to improve pain and function. However, its effect on postoperative sleep quality remains poorly defined. The purpose of this systematic review is to summarize changes in sleep quality after shoulder arthroplasty.

**Methods:**

MEDLINE, Embase and Web of Science were searched from database inception to 31 October 2025, for studies reporting postoperative sleep outcomes following primary shoulder arthroplasty with at least one reported postoperative sleep-related outcome. Due to heterogeneity amongst studies and outcomes, results were synthesized descriptively.

**Results:**

Eighteen studies comprising 2512 patients were included. Preoperative sleep disturbance was highly prevalent (84–97%). Minimal improvement in sleep quality was reported within the first 6 weeks postoperatively, followed by a marked improvement by 6 months. This was generally sustained at longer follow-up. However, 13–16% of patients reported persistent difficulties sleeping comfortably. Seventeen studies (94%) reported subjective sleep outcomes, while only two studies (11%) reported on objective sleep measures using wearable devices.

**Conclusion:**

Shoulder arthroplasty is generally associated with meaningful improvement in sleep quality between 6 weeks and 6 months postoperatively, with improvements plateauing thereafter. The existing literature is limited by heavy reliance on subjective, often non-validated, sleep measures and a paucity of objective sleep data.

## Introduction

Sleep disturbance is highly prevalent amongst patients with shoulder pathology and represents a significant source of disability. Mulligan et al. reported that 75% of the subacromial impingement syndrome group, 71% of the rotator cuff tear group, 85% of the osteoarthritis group and 86% of the adhesive capsulitis group met their criteria for poor sleep quality, demonstrating that sleep disturbance is common across a broad spectrum of shoulder conditions.^1^ Patients with these conditions experience nocturnal pain, difficulty finding a comfortable sleeping position and frequent awakenings, which impair both sleep initiation and maintenance. Poor sleep has been shown to contribute to worse pain perception, delayed functional recovery and reduced quality of life.^2–4^ As a result, improving sleep quality has become an increasingly important treatment goal in the management of shoulder disorders.

In recent years, shoulder arthroplasty has become increasingly common amongst patients with a variety of shoulder pathologies.^5^ Both anatomic and reverse total shoulder arthroplasty (aTSA and rTSA, respectively) are now well-established treatments for glenohumeral arthritis, rotator cuff tear arthropathy and complex proximal humerus fractures, with demonstrated improvements in pain relief and shoulder function.^6^ Despite the growing volume of shoulder arthroplasty procedures, sleep outcomes in this population remain relatively under-studied. Two recent systematic reviews examining sleep disturbance after shoulder surgery included only a small subset of studies evaluating sleep quality specifically following shoulder arthroplasty and reported overall short- and long-term improvements in sleep quality following surgery.^7,8^ However, shoulder arthroplasty outcomes were not the primary focus in either review, and sleep-related findings specific to arthroplasty were not synthesized. Unlike arthroscopic shoulder procedures, shoulder arthroplasty is typically performed in an older population with chronic degenerative pathology and results in substantial alterations in glenohumeral anatomy and biomechanics, all of which may uniquely influence postoperative sleep quality and recovery.

To our knowledge, no prior review has comprehensively synthesized the available evidence examining the impact of shoulder arthroplasty on sleep quality. Therefore, the purpose of this systematic review is to summarize changes in sleep quality after shoulder arthroplasty, compare available subjective and objective sleep assessment tools and identify gaps in the existing literature to inform future research.

## Methods

### Search strategy

A comprehensive literature search was conducted across MEDLINE (Ovid), Embase (Ovid) and Web of Science Collection from database inception to 31 October 2025. The strategy was developed a priori and adhered to the Preferred Reporting Items for Systematic Reviews and Meta-Analyses (PRISMA) 2020 guidelines. Search terms combined controlled vocabulary and free text keywords representing two core concepts: i) any type of shoulder arthroplasty and ii) sleep quality or sleep disturbance. Boolean operators, truncation and proximity commands were used to maximize sensitivity. The full search strategies are provided in Supplementary Table 1. No date restrictions were applied at the search stage to maximize sensitivity. Reference lists of included studies and relevant reviews were manually screened to identify additional publications.

### Eligibility criteria

Studies were eligible for inclusion if they met the following criteria: i) patients ≥18 years of age who underwent a primary shoulder hemiarthroplasty, aTSA or rTSA for any surgical indication, and ii) report of at least one quantitative or qualitative postoperative measure of sleep. Exclusion criteria included: i) non-arthroplasty shoulder procedures, ii) no postoperative sleep-related outcome reports, iii) abstracts, case study, systematic review and meta-analyses, iv) unable to retrieve full text and v) studies not written in English (excluded at the abstract review stage).

### Study selection

**Figure 1.**
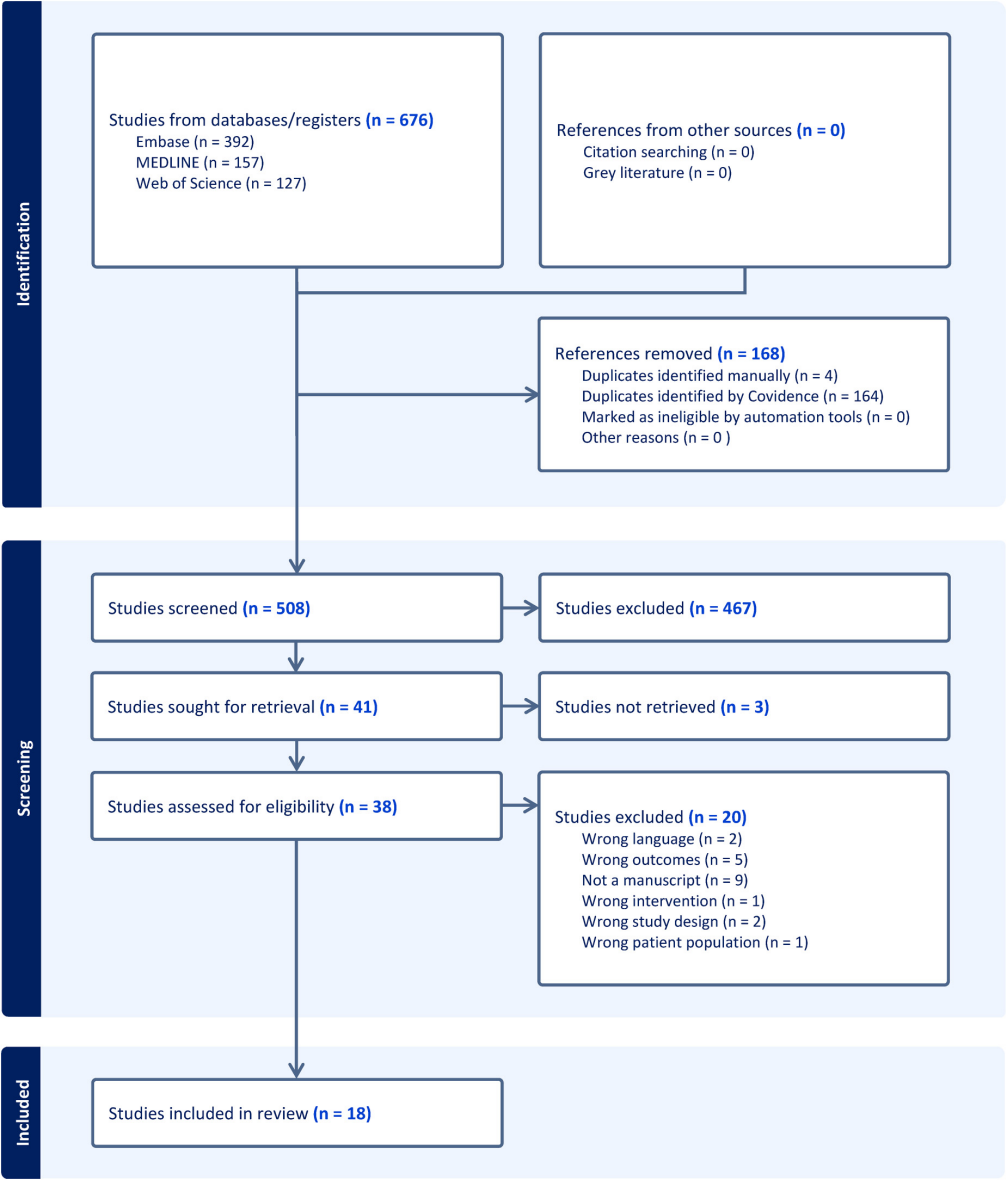
PRISMA flow diagram.

All records were imported into Covidence (Veritas Health Innovation, Melbourne, Australia) for screening and management. Two reviewers independently screened titles and abstracts to identify studies meeting the inclusion criteria. Full texts were then reviewed by both authors, and any disagreements were resolved by consultation with a third reviewer. The study selection process and reasons for exclusion are included in Figure 1.

### Data extraction

Data extraction was performed independently by two reviewers using a standardized, piloted form. Extracted information included study characteristics (first author, year of publication), participant demographics (age, sex, indication for arthroplasty), surgical details (hemiarthroplasty, aTSA or rTSA), sleep assessment tools, timing of evaluation and reported outcomes. For studies enrolling mixed surgical populations, only data pertaining to the shoulder arthroplasty subgroup were extracted and included in this review.

### Risk of bias and quality assessment

Methodological quality was assessed independently by two reviewers. The Cochrane Collaboration's Risk of Bias Tool 2 (RoB 2) was used to assess randomized trials.^9^ Each domain was rated as low risk, some concerns or high RoB, with discrepancies resolved by consensus. The quality of non-randomized studies was measured using the Methodological Index for Non-Randomized Studies (MINORS) score.^10^ This is a 12-item instrument that was designed to assess the methodological quality of comparative and non-comparative non-randomized studies. For each article, each item was scored according to a 3-point scale: 0 (not reported), 1 (inadequately reported) or 2 (adequately reported). Four items (9–12) are only relevant to comparative studies. As such, comparative studies can score a maximum of 24 points, whereas non-comparative studies can score a maximum of 16 points. A higher score correlates with a higher quality study. For non-comparative studies, a score of ≤8 was considered poor quality, 9–14 considered moderate quality and ≥15 high quality. For comparative studies, ≤14 was considered poor, 15–22 moderate and ≥23 high quality.^11^

### Statistical analysis

Due to the lack of standardized outcomes across studies, no statistical comparisons or meta-analyses were performed, and therefore, the analyses were primarily descriptive.

## Results

### Search results

The search strategies from Web of Science, Embase and MEDLINE retrieved 676 articles (Figure 1). After automatic removal of duplicates and following screening based on title and abstract, 39 full-text articles were assessed for eligibility according to the inclusion and exclusion criteria. Following exclusions, 18 were included in this systematic review. Both included RCTs were judged to have an overall RoB of some concern, with low risk in the domains of randomization, missing outcome data and selective reporting.^12,13^ The main sources of potential bias were related to deviations from intended interventions and reliance on subjective patient-reported outcomes, resulting in minor performance and detection bias. The average MINORS score was 15.7 out of 24 across comparative studies and 10.4 out of 16 across non-comparative studies.

### Study characteristics

A total of 2512 patients, with individual study sample sizes ranging between 14 and 989, were included in this study. Mean patient age ranged between 60.6 and 82.0 years, and males accounted for 16.7–70% of participants across studies. From these, 745 patients underwent aTSA, 1037 rTSA, 63 hemiarthroplasty and 493 were unspecified between aTSA and rTSA. Of note, two studies reported mixed aTSA and rTSA cohorts without specifying the individual breakdown.^14,15^ Indications for surgery included glenohumeral osteoarthritis (*n* = 7 studies), rotator cuff arthropathy (*n* = 6 studies), rheumatoid arthropathy (*n* = 2 studies), avascular necrosis (*n* = 2 studies), failed previous arthroplasty (*n* = 2 studies), proximal humerus fractures (*n* = 2 studies), massive rotator cuff tear without arthritis (*n* = 1 study), post-traumatic arthritis (*n* = 1 study) and malunion (*n* = 1 study). Of note, two studies excluded patients with proximal humerus fractures. Five studies did not provide any information regarding surgical indications (Table 1).

**Table 1.** Study characteristics.

Author, year	*n*	Male (%)	Age (years)	Surgery	Surgical indication
Cheah et al. 2022	125	51	66.1 ± 10.1	aTSA (*n* = 48) rTSA (*n* = 75) Hemiarthroplasty (*n* = 2)	OA, PTA, RA, RCA
Gadangi et al. 2023	20	55	65.7 ± 8.1	aTSA (n not specified) rTSA (n not specified)	OA
Chua et al. 2020	70	34	75.0 ± 10.0	rTSA	RCA, RA, Fracture, Failed Arthroplasty, Massive RTC, AVN
George et al. 2022	223	nr	nr	TSA	nr
Ilfeld et al. 2005	14	57	64.6 ± 10.6	TSA	OA, Failed arthroplasty
Ilyas et al. 2024	108	27	68.0 ± 5.1	rTSA	RCA
Kaymakoglu et al. 2025	46	46	68.5 ± 10.8	rTSA	RCA
Kolade et al. 2023	154	49	67.0 ± 10.0	aTSA (n not specified) rTSA (n not specified)	Excluded if done for fracture
Macfarlane et al. 2024	93	48	60.6 ± 8.9	aTSA (*n* = 45) rTSA (*n* = 48)	OA, RCA, AVN, Malunion
Matsen et al. 1996	29	69	65.0 ± 13.0	TSA	OA
Matsen et al. 2019	43	55	71.0 ± 10.0	Hemiarthroplasty	RCA
Nasr et al. 2024	100	49	69.6 ± 8.0	rTSA	nr
Norman et al. 2024	227	55	67.0 ± 9.7	TSA	nr
O'Donell et al. 2025	107	57	65.0 ± 9.1	aTSA (*n* = 58) rTSA (*n* = 49)	OA, RCA, Fracture sequelae
Pai et al. 2025	72	39	69.6 ± 7.3	aTSA (*n* = 35) rTSA (*n* = 37)	Not reported
Vegas et al. 2023	989	50	71.9 ± 6.7	aTSA (*n* = 517) rTSA (*n* = 472)	Not reported
Weinberg et al. 2020	74	46	65.8 ± 12.5	aTSA (*n* = 42) rTSA (*n* = 32)	OA
Wretenberg et al. 1997	18	17	82.0 ± 5.5	Hemiarthroplasty	3- or 4-part proximal humerus fractures

*n* refers to sample size. nr refers to not reported. Age reported as mean ± standard deviation. aTSA and rTSA refer to anatomic and reverse total shoulder arthroplasty, respectively. TSA refers to total shoulder arthroplasty; used in cases where no distinction was made between aTSA and rTSA. OA: osteoarthritis; PTA: post-traumatic arthritis; RA: rheumatoid arthritis; RCA: rotator cuff arthropathy; AVN: avascular necrosis.

### Sleep quality outcomes

Of the 18 included studies, 17 studies (94%) reported at least one subjective sleep-related outcome (Table 2). Amongst the 17 studies that assessed subjective sleep outcomes, the Pittsburgh Sleep Quality Index (PSQI) was the most commonly used (*n* = 5 studies; 28%) followed by the PROMIS sleep disturbance short forms (*n* = 2 studies; 11%), the Leeds Sleep Evaluation Questionnaire (LSEQ, *n* = 1 study, 6%) and the Jenkins Sleep Scale (JSS, *n* = 1, 6%). In four studies (24%), sleep disturbance was assessed indirectly using broader functional questionnaires (e.g., Simple Shoulder Test or ASES-derived items) that included sleep-related questions rather than dedicated sleep instruments. A total of seven studies (41%) used non-validated or study-specific Likert-scale measures, most commonly evaluating sleep quality, sleep duration, nocturnal pain–related sleep disturbance or difficulty initiating or maintaining sleep. Twelve studies (67%) reporting subjective outcomes included a preoperative baseline sleep assessment; however, one reported the exact preoperative timing of this measurement (Table 3). The timing of postoperative sleep assessment varied considerably. Early postoperative assessments (≤2 weeks) were reported in 5 studies (29%). Intermediate follow-up (6 weeks to 6 months) was assessed in 11 studies (65%), while long-term follow-up (≥12 months) was reported in seven studies (41%), including two studies with follow-up extending beyond 2 years. Most studies reported multiple postoperative time points.

**Table 2.** Studies evaluating subjective sleep quality outcomes.

Study	Surgery	Sleep quality measure	Timepoints	Results
Cheah et al. 2022	aTSA rTSA HA	LSEQ, Sleep diaries	Pre op: Not specified Post op: POD 10–14	Patients in the intervention group reported improved inpatient sleep quality on the LSEQ, though not across all domains. No differences were observed at the POD 10–14 follow up.
Chua et al. 2020	rTSA	Likert scale evaluating i) frequency of pain during sleep; ii) severity of pain during sleep	Pre op: Not specified Post op: 1, 6, 12, 24 weeks	At 6 weeks, pain during sleep decreased from daily to between monthly to weekly for both groups. At 24 weeks, both groups had significant reduction in the frequency and severity of their pain during sleep.
George et al. 2022	TSA	PROMIS short form for sleep disturbance	Post op: 6–60 months	There was a difference in sleep disturbance by chronic pain grade, with better outcomes associated with lesser chronic pain grades in TSA.
Ilfeld et al. 2005	TSA	Number of i) patients reporting difficulties sleeping; ii) awakening for each patient	Post op: POD 0–6	The number of patients who reported issues sleeping postop decreased from 2 on POD0–1 on POD6. Median awakenings per patient were 0 from POD0 to POD6.
Ilyas et al. 2024	rTSA	PSQI	Pre op: Not specified Post op: 6 weeks, 6 and 12 months	No significant change was observed between preoperative and 6-week assessments, a significant improvement occurred between 6 weeks and 6 months, and no further difference was seen between 6 months and 1 year.
Kaymakoglu et al. 2025	rTSA	PSQI, JSS	Pre op: Not specified Post op: 1, 3, 6, 12 months and latest follow up	PSQI scores decreased significantly by 3 months and remained stable during subsequent follow-ups. JSS scores showed a significant reduction from preoperative levels to the first postoperative month with no significant changes thereafter. The proportion of patients with clinically significant sleep disturbance declined from 45.7% preoperatively to 26.1% by 3 months and remained stable at later follow ups.
Kolade et al. 2023	aTSA rTSA	Likert Scale evaluating difficulty falling asleep, interrupted sleep and reduced sleep	Pre op: Not specified Post op: 2, 6, 12 weeks	Preoperatively 13% of patients complained of sleep disturbances as primary complaint, and this number peaked 2 weeks postoperatively to 33% and then decreased to 26% at 6 weeks and 21% at 3 months. The sleep score demonstrated gradual improvement postoperatively compared to preoperative score with 72% improvement at 6-week time point and 104% improvement at 3-month time point.
Macfarlane et al. 2024	aTSA rTSA	Likert scale of sleep duration and sleep quality	Post op: POD 0–30	Pain catastrophizing scale scores were predictive of shorter sleep duration and worse sleep quality.
Matsen et al. 1996	TSA	SST	Post op: mean follow up 303 ± 164 days	4 (14%) of patients were able to sleep on the operative side preoperatively, 25 (86%) patients were able to do so postoperatively.
Matsen et al. 2019	HA	SST	Pre op: Not specified Post op: 2 years	The percentage of patients able to sleep comfortably increased from 19% (8 of 42) to 71% (30 of 42).
Nasr et al. 2024	rTSA	PSQI	Pre op: Not specified Post op: 6, 12 months	Average PSQI scores improved in both groups. However, 33 patients showed no change in PSQI or had worse sleep at 1 year post surgery.
Norman et al. 2024	TSA	PROMIS 6-item short forms for sleep disturbance	Post op: at least 6 months	PROMIS measures of sleep disturbance were associated with poorer outcomes in individuals with high impact chronic pain.
O'Donnell et al. 2025	aTSA rTSA	PSQI; VAS - Quality of Sleep	Pre op: Not specified Post op: 2, 6, 12, 24 weeks	Both aTSA and rTSA were associated with significant and sustained postoperative improvements in sleep quality, with meaningful gains evident by 6 weeks and continuing through 6 months.
Pai et al. 2025	aTSA rTSA	Sleep disturbances as mild, moderate or severe	Post op: POD 1 and 2	No differences in sleep quality or satisfaction were seen between the treatment groups at postoperative day 2.
Vegas et al. 2023	aTSA rTSA	SST; ASES	Pre op: Not specified Post op: 3, 6, 12 months and latest follow up	Significant improvements in the SST and ASES sleep questions were observed from preoperatively to 3 months in both groups. The ability to sleep comfortably reached a plateau at 3 months and the ability to sleep on the affected side reached a plateau at 6 months.
Weinberg et al. 2020	aTSA rTSA	PSQI	Pre op: Not specified Post op: 6, 12, 24, 48 weeks	The PSQI score significantly improved at 6 weeks. PSQI scores decreased until the 12 month follow up, although changes were not statistically significant.
Wretenberg et al. 1997	HA	Are you able to sleep on the operated side?	Post op: mean 3.5 (2–7) years	At the time of the follow up, all patients could sleep on the operated side.

aTSA and rTSA refer to anatomic and reverse total shoulder arthroplasty, respectively. TSA refers to total shoulder arthroplasty; used in cases where no distinction was made between aTSA and rTSA. HA: hemiarthroplasty; LSEQ: Leeds Sleep Evaluation Questionnaire; PSQI: Pittsburgh Sleep Quality Index; JSS: Jenkins Sleep Scale; SST: Simple Shoulder Test; ASES: American Shoulder and Elbow Surgeons questionnaire; PROMIS: Patient-Reported Outcome Measurement Information System measures; VAS: Visual Analog Scale; Pre op and Post op: the preoperative and postoperative time periods, respectively; POD: postoperative day.

**Table 3.** Studies reporting pre- and postoperative sleep quality outcomes.

Study	Surgery	Sleep quality measure	Pre op	Post op	Results
Cheah et al. 2022	aTSA rTSA HA	LSEQ, Sleep diaries	NS	10–14 days	Patients in the intervention group reported improved inpatient sleep quality on the LSEQ, though not across all domains. No differences were observed at the POD 10–14 follow up.
Gadangi et al. 2023	aTSA rTSA	Actigraphy	34 days	0–2; 2–4; 4–6 weeks	Deep sleep decreased in the first 0–2 weeks. Total sleep time increased at 2–4 and 4–6 weeks postoperatively. REM sleep increased postoperatively at 2–4 weeks.
Chua et al. 2020	rTSA	Likert scale: pain frequency/severity during sleep	NS	1, 6, 12, 24 weeks	At 6 weeks, pain during sleep decreased from daily to between monthly to weekly for both groups. At 24 weeks, both groups had significant reduction in the frequency and severity of their pain during sleep.
Ilyas et al. 2024	rTSA	PSQI	NS	6, 26, 52 weeks	No significant change was observed between preoperative and 6 week assessments, a significant improvement occurred between 6 weeks and 6 months, and no further difference was seen between 6 months and 1 year.
Kaymakoglu et al. 2025	rTSA	PSQI, JSS	NS	1, 3, 6, 12 months and latest follow up	PSQI scores decreased significantly by 3 months and remained stable during subsequent follow-ups. JSS scores showed a significant reduction from preoperative levels to the first postoperative month with no significant changes thereafter. The proportion of patients with clinically significant sleep disturbance declined from 45.7% preoperatively to 26.1% by 3 months and remained stable at later follow ups.
Kolade et al. 2023	aTSA rTSA	Likert Scale evaluating difficulty falling asleep, interrupted sleep and reduced sleep	NS	2, 6, 12 weeks	Preoperatively 13% of patients complained of sleep disturbances as primary complaint, and this number peaked 2 weeks postoperatively to 33% and then decreased to 26% at 6 weeks and 21% at 3 months. The sleep score demonstrated gradual improvement postoperatively compared to preoperative score with 72% improvement at the 6 week time point and 104% improvement at the 3 month time point.
Matsen et al. 1996	TSA	SST	NS	Mean follow up 303 ± 164 days	4 (14%) of patients were able to sleep on the operative side preoperatively, 25 (86%) patients were able to do so postoperatively.
Matsen et al. 2019	HA	SST	NS	2 years	The percentage of patients able to sleep comfortably increased from 19% (8 of 42) to 71% (30 of 42).
Nasr et al. 2024	rTSA	PSQI	NS	6, 12 months	Average PSQI scores improved in both groups. However, 33 patients showed no change in PSQI or had worse sleep at 1 year post surgery.
O'Donnell et al. 2025	aTSA rTSA	PSQI; VAS - Quality of Sleep	NS	2, 6, 12, 24 weeks	Both aTSA and rTSA were associated with significant and sustained postoperative improvements in sleep quality, with meaningful gains evident by 6 weeks and continuing through 6 months.
Vegas et al. 2023	aTSA rTSA	SST; ASES	NS	3, 6, 12 months and latest follow up	Significant improvements in the SST and ASES sleep questions were observed from preoperatively to 3 months in both groups. The ability to sleep comfortably reached a plateau at 3 months and the ability to sleep on the affected side reached a plateau at 6 months.
Weinberg et al. 2020	aTSA rTSA	PSQI	NS	6, 12, 24, 48 weeks	The PSQI score significantly improved at 6 weeks. PSQI scores decreased until the 12 month follow up, although changes were not statistically significant.

aTSA and rTSA: anatomic and reverse total shoulder arthroplasty, respectively; HA: hemiarthroplasty; NS: not specified – used in studies where preoperative values were reported; however, the time at which they were taken was not specified; LSEQ: Leeds Sleep Evaluation Questionnaire; PSQI: Pittsburgh Sleep Quality Index; JSS: Jenkins Sleep Scale; SST: Simple Shoulder Test; ASES: American Shoulder and Elbow Surgeons questionnaire; VAS: Visual Analog Scale; Pre op and Post op: the preoperative and postoperative time periods, respectively; REM: rapid eye movement.

Objective sleep outcomes were assessed in 2 of 18 included studies (11%), both using wearable sleep-tracking devices, with only one of them reporting a preoperative objective sleep assessment. Cheah et al. used wrist-worn actigraphy during the first 24 h postoperatively and reported that patients managed with an interventional multimodal sleep pathway demonstrated greater total sleep time, fewer minutes awake and higher sleep efficiency compared with those receiving standard care.^12^ Gadangi et al. employed the WHOOP 2.0 wearable device to assess total sleep time and sleep stage distribution, including REM, deep and light sleep, through 6 weeks postoperatively. They observed an early postoperative reduction in deep sleep during the 0–2 week interval relative to baseline, followed by increased total sleep time at the 2–4 and 4–6 week intervals compared with preoperative values.^14^

### Changes in sleep quality following surgery

Across the 18 included studies, only 12 studies provided a comparison of sleep quality measurements pre- and postoperatively (Table 3). Sleep disturbance was highly prevalent preoperatively, affecting 84–97% of patients.^16–18^ There were mixed results between studies regarding improvements in sleep quality by 6 weeks, with some reporting decreases,^14,15,19^ whereas others reported no change.^16,18,20^ However, a marked increase in sleep quality was observed between 6 weeks and 6 months postoperatively, which was sustained throughout the remainder of follow-ups across all studies. Interestingly, recovery of sleep quality followed distinct trajectories, with the ability to sleep comfortably improving rapidly and plateauing by 3 months, whereas the ability to sleep on the operative side improved more gradually and continued through 6 months.^17^

### Persistent post-operative sleep disturbance

Despite an overall improvement in sleep quality postoperatively, 13–16% of patients report continued difficulty sleeping comfortably, 31–37% of patients remain unable to sleep on the operative side, and a subset of patients report persistent or worsening sleep quality at one year following surgery.^17,20,21^

## Discussion

This systematic review aimed to provide a comprehensive synthesis of the literature exploring the effect of shoulder arthroplasty on sleep quality. The main finding of this review is that sleep quality generally improves between the 6-week and 6-month postoperative timeframe. However, the existing literature is mostly reliant on non-validated subjective sleep quality measures, with objective sleep outcomes being infrequently assessed. While most studies suggest clinically meaningful postoperative improvements in patient-reported sleep disturbance, the magnitude, timing and physiological correlates of these improvements remain poorly defined.

Despite the reported improvement in sleep quality by the 6-month postoperative time point, a subset of patients show no improvement, while others report worsening sleep quality postoperatively.^17,20,21^ These findings underscore the importance of identifying patients at high risk for persistent postoperative sleep disturbance and implementing targeted perioperative interventions. Nasr et al. examined potential predictors in this population and found no association between elevated neutrophil-to-lymphocyte ratios (≥2.5) and PSQI scores at 6 or 12 months postoperatively.^21^ While no studies in this review evaluated risk factors for sleep disturbance after shoulder arthroplasty, George et al. noted that chronic pain was associated with poor sleep quality in patients who had undergone shoulder arthroplasty.^22^ This is consistent with prior work in total hip arthroplasty and spine surgery populations, which identified pain, anxiety, preoperative sleep disorder and VAS scores as significant contributors to early postoperative sleep disturbance.^23,24^ However, further research is needed to provide additional insight into risk factors for postoperative sleep disturbance in patients undergoing shoulder arthroplasty.

Only eight studies used a validated sleep questionnaire such as the PSQI, JSS, LSEQ and PROMIS-6. Several studies utilized specific questions from the SST,^17,25,26^ whereas others have used Likert scales of different sleep quality measures.^15,27^ Studies relying solely on SST or ASES sleep items should be interpreted with caution, as these instruments contain only a single sleep-related question each, reflecting nocturnal pain rather than the broader, multi-dimensional nature of sleep quality. Using validated sleep questionnaires is essential as they safeguard reliability, validity and responsiveness, which ensures the measured changes are a true representation of patient experience. Sleep quality is a multifactorial construct that is difficult to define and measure objectively, even with validated sleep questionnaires, all of which have their strengths and limitations.^28^ Future work using subjective sleep quality measures should be cautious of the measures taken to provide reliable and valid findings that can be compared against the existing literature.

In addition to validated questionnaires, few studies have utilized available technologies to objectively quantify sleep quality. Previous work has demonstrated differences in the mean of self-reported and measured sleep, with subjective reports averaging almost an hour greater than measured sleep.^29^ This may reflect the fact that subjective sleep reports may suffer from recall bias, thus affecting patient-reported outcomes. To address these limitations, objective sleep-assessment technologies, including polysomnography and wrist actigraphy, have been increasingly incorporated into sleep research. Wrist actigraphy involves the use of a portable device that records movement over extended periods of time and has been used extensively in the study of sleep and circadian rhythms. In 2007, the American Academy of Sleep Medicine supported the use of actigraphy for clinical application.^30^ Despite this, only two studies use validated objective measures of sleep quality up to 6 weeks postoperatively.^12,14^

This systematic review has several limitations that warrant consideration. First, the overall quality of the included studies was variable, with many exhibiting suboptimal study designs and small sample sizes, limiting statistical power and increasing susceptibility to bias. Second, there was substantial heterogeneity in outcome assessment, including inconsistent use of non-validated and mostly subjective assessments of sleep quality in shoulder arthroplasty research. Although the lack of objective sleep quality outcomes is a limitation of the current literature investigating sleep quality in shoulder arthroplasty, it is worth noting that subjective and objective measures capture different aspects of sleep.^31,32^ As such, literature aiming to provide meaningful clinical assessments of sleep quality should aim to incorporate both sets of measurements.

## Conclusion

This systematic review demonstrates that shoulder arthroplasty is generally associated with improvements in sleep quality, with the most consistent gains occurring between 6 weeks and 6 months postoperatively and plateauing thereafter. Although most patients report improved sleep, a clinically relevant subset continues to experience persistent sleep disturbance at long-term follow-up, underscoring heterogeneity in recovery trajectories. Emerging evidence suggests that perioperative strategies targeting pain and physiologic stress may mitigate early postoperative sleep disruption. However, these conclusions are constrained by the heavy reliance on subjective, often non-validated sleep measures, limited longitudinal pre–post comparisons and a paucity of objective sleep data. Future studies should incorporate validated subjective instruments alongside objective sleep assessments across standardized time points to better characterize sleep recovery, identify patients at risk for persistent disturbance and inform targeted perioperative and postoperative interventions.

## Supplemental Material

sj-docx-1-sel-10.1177_17585732261450975 - Supplemental material for Sleep quality after shoulder arthroplasty – A systematic review Supplemental material, sj-docx-1-sel-10.1177_17585732261450975 for Sleep quality after shoulder arthroplasty – A systematic review by Kalter Hali, Marc A Manzo, Sean Mckellar, Matthew J Raleigh and Ujash Sheth in Shoulder & Elbow
