# Supplementary material for: Sleep quality after shoulder arthroplasty – A systematic review
Source: Shoulder Elbow. 2026 May 23:17585732261450975. Online ahead of print. doi: 10.1177/17585732261450975 (PMC13198522; doi:10.1177/17585732261450975)
Supplement: sj-docx-1-sel-10.1177_17585732261450975 - Supplemental material for Sleep quality after shoulder arthroplasty – A systematic review [file sj-docx-1-sel-10.1177_17585732261450975.docx]

**Supplementary Table 1.** Search strategy

| **Medline** | |
| --- | --- |
| 1. exp Arthroplasty, Replacement, Shoulder/ 2. (shoulder adj3 (arthroplast* or replac* or prosthe* or hemiarthroplast* or "total shoulder" or "reverse shoulder")).ti,ab,kw. 3. (TSA or RSA).ti,ab. 4. exp Sleep/ 5. (sleep adj3 (qualit* or disturb* or impair* or disorder* or problem* or loss or pattern* or effic* or position or posture or "sleep latency" or "sleep onset")).ti,ab,kw. 6. insomnia.ti,ab,kw. 7. ("Pittsburgh Sleep Quality Index" or PSQI).ti,ab,kw. 8. ("Leeds Sleep Evaluation Questionnaire" or LSEQ).ti,ab,kw. 9. (actigraph* or (wearable adj3 (device* or monitor* or actimetr*))).ti,ab,kw. 10. 1 or 2 or 3 11. 4 or 5 or 6 or 7 or 8 or 9 12. 10 and 11 | |
| **EMBASE** | |
| 1. exp Arthroplasty, Replacement, Shoulder/ 2. (shoulder adj3 (arthroplast* or replac* or prosthe* or hemiarthroplast* or "total shoulder" or "reverse shoulder")).ti,ab,kw. 3. (TSA or RSA).ti,ab. 4. exp Sleep/ 5. (sleep adj3 (qualit* or disturb* or impair* or disorder* or problem* or loss or pattern* or effic* or position or posture or "sleep latency" or "sleep onset")).ti,ab,kw. 6. insomnia.ti,ab,kw. 7. ("Pittsburgh Sleep Quality Index" or PSQI).ti,ab,kw. 8. ("Leeds Sleep Evaluation Questionnaire" or LSEQ).ti,ab,kw. 9. (actigraph* or (wearable adj3 (device* or monitor* or actimetr*))).ti,ab,kw. 10. 1 or 2 or 3 11. 4 or 5 or 6 or 7 or 8 or 9 12. 10 and 11 | |
| **Web of Science** | |
|  | TS=( (shoulder AND (arthroplast* OR replac* OR prosthe* OR "reverse shoulder" OR "total shoulder" OR TSA OR RSA)) AND (sleep OR "sleep quality" OR "sleep disturbance" OR insomnia OR PSQI OR actigraph* OR wearable*) ) |
